# Reducing inequities in maternal and child health in rural Guatemala through the CBIO+﻿ Approach of Curamericas: 9.﻿ Key stakeholder perspectives on strengthening the CBIO+ Approach

**DOI:** 10.1186/s12939-022-01761-x

**Published:** 2023-02-28

**Authors:** Jason Lambden, Shayanne Martin, Mario Valdez, Ira Stollak, Carey C. Westgate, Henry B. Perry

**Affiliations:** 1 McGaw Medical Center, Northwestern University, Chicago, Illinois USA; 2 Institute for Global Health Sciences, University of California San Francisco, San Francisco, California USA; 3 Curamericas/Guatemala, Calhuitz, San Sebastián Coatán, Huehuetenango, Guatemala; 4 Curamericas Global, Raleigh, North Carolina USA; 5 Community Health Impact Coalition, New York, New York USA; 6 Health Systems Program, Department of International Health, Johns Hopkins Bloomberg School of Public Health, Baltimore, Maryland USA

**Keywords:** Maternal health, Child health, Community health, Primary health care, Community-based primary health care, Implementation research, Census-Based, Impact-Oriented Approach, Care Groups, Community birthing centers, Guatemala, Equity, Curamericas Global, Curamericas/Guatemala

## Abstract

**Background:**

Community-based health interventions have been an integral part of recent health gains globally. An innovative approach to delivering community health care combines the Census-Based, Impact-Oriented (CBIO) Approach with Care Groups and Community Birthing Centers called *Casas Maternas Rurales*. CBIO+ was adopted by Curamericas/Guatemala in its Maternal and Child Health Project, 2011–2015. Here, we describe the opinions of Project staff and local government health care workers about the strengths and challenges of CBIO+.

**Methods:**

Self-administered questionnaires, key informant interviews, and focus group discussions were used to obtain the views of 21 staff members from Curamericas/Guatemala as well as 15 local government health workers. The evaluation focused on four primary areas: (1) advisability of integrating the CBIO+ Approach into the government’s rural health system, (2) staff knowledge of the CBIO+ Approach, (3) advantages, disadvantages and challenges of the CBIO+ Approach, and (4) proposed improvements to the CBIO+ Approach. The data were coded into categories and from these categories themes were derived.

**Results:**

The most commonly mentioned advantage of CBIO+ was the inclusion of the community in program planning, which improved participation. Many respondents noted that the CBIO+ Approach was challenging to implement in communities with internal conflicts. Among other challenges mentioned were coordinating (both among the Project staff and with others in the communities), maintenance of a high level of community participation, and overcoming opposition of men to women’s participation in Care Groups. The staff mentioned a number of possible changes, including increasing male involvement, raising salaries for community-level paid staff, providing volunteers with incentives, and improving coordination both internally and externally. There was a strong demand among the local Ministry of Public Health and Social Welfare staff for the Project to continue.

**Conclusion:**

The CBIO+ Approach and its implementation by Curamericas/Guatemala was overall embraced by local staff. By eliciting﻿ feedback while the project was ongoing, actionable areas for improvement were identified.

## Background

This is the ninth article in a series of 10 papers describing the effectiveness of a Maternal and Child Health Project implemented by Curamericas/Guatemala from 2011–2015 (hereafter referred to as the Project) which utilized the Expanded Census-Based, Impact-Oriented (CBIO+) Approach in improving maternal and child health in an isolated mountainous region of northwest Guatemala. The term CBIO+ Approach refers to the expansion of the Census-Based, Impact-Oriented Approach to include Care Groups and *Casas Maternas Rurales* (literally Rural Maternal Homes, which are referred to hereafter as Community Birthing Centers).

The first eight papers describe the Project and its evaluation methodology [1, 2], the expansion of coverage of key maternal and child survival interventions in the Project’s catchment area [3], improvements in nutritional status of children [4], and mortality impact [5]. Paper 6 [6] presents an evaluation of the quality of care provided at Community Birthing Centers. Papers 7 and 8 focus on empowerment: Paper 7 concerns the empowerment of women who worked as Care Group Volunteers and the strengthening of social capital in the Project Area that occurred as a result of the Project [7], and Paper 8 addresses the empowerment of women in the Project Area that is attributable to Project activities [8].

In this paper we describe the results of implementation research designed to identify the strengths and weaknesses of the CBIO+ Approach and its implementation as viewed by Project staff members and their counterparts in the local government health services. Key staff were interviewed about their perspectives, beliefs and knowledge of the CBIO+ Approach as it was implemented by the Project along with strengths, weaknesses and specific recommendations for improvement, with a particular focus on how the Project might be integrated into Guatemala’s national rural health system, including its *Programa de Extensión do Cobertura* (PEC, Extension of Coverage Program).

From 1997 to 2014, the Government of Guatemala’s *Ministerio de Salud Pública y Asistencia Social* (Ministry of Public Health and Social Welfare, or MSPAS) contracted with local non-governmental organizations (NGOs) through its PEC Program to extend the provision of preventive and curative health services to rural indigenous communities isolated from public health services [9, 10]. The four main services covered by the PEC’s basic health package were: (1) comprehensive health care for women; (2) health care for children; (3) infectious diseases and emergency care, including for accidents; and (4) environmental care covering vector control, proper waste disposal, water quality, food safety, and home hygiene [9]. PEC’s community outreach program for isolated rural areas was called *Sistema Integral de Atención en Salud* (SIAS, Integrated System of Health Care). Through SIAS, NGOs in isolated rural areas received funding from the government to hire a health team consisting of a doctor or nurse (in practice, nearly always a nurse) who worked in coordination with *Facilitadoras Comunitarias* (Community Facilitators who were part of the PEC Program). Community Facilitators were responsible for assisting the doctor or nurse during his or her monthly visits to communities to provide immunizations, prenatal care, family planning services, and treatment of children with acute illnesses. Services were provided at MSPAS health posts, rural mini-clinics that were not permanently staffed, and *Centros de Convergencia* (Meeting Centers), which were public facilities (such as schools) where various services were provided, including care from the PEC mobile team. The PEC/SIAS Program sent nurses to visit each community at least once a month to provide health services.

During this time period, the Project adapted its CBIO+ Approach to include pre-existing Community Facilitators as trainers of Care Group Volunteers and collectors of vital events data. In this paper and in the other papers in this series, we refer to these Community Facilitators working in the Project as Level-1 Care Group Promoters in order to better link our work with the broader Care Group literature, which recognizes that the role of a Promoter is to meet with Care Groups to teach health promotion messages [11]. Curamericas/Guatemala received funds from MSPAS to operate the PEC program in the municipalities of San Sebastián Coatán and San Miguel Acatán, and the Guatemalan NGO *Asociación de Desarollo Integral de Vida y Esperanza* (the Association for Integrated Development of Life and Hope, or ADIVES) provided PEC in the municipality of Santa Eulalia. The Birthing Centers, like PEC, also fulfilled demand, primarily for maternal/newborn services.

The Project was implemented with the explicit aim of integrating PEC and CBIO+ to create a new, even more effective model of an integrated community-based rural health system that the Project staff likened to a “table with four legs,” namely CBIO, Care Groups, the Birthing Centers, and PEC. Care Groups helped to change key household health behaviors and generate demand for health services [4]. PEC helped to fulfill this demand at the community level by bringing basic health services such as antenatal care, treatment of diarrhea and acute respiratory infection, and immunizations for pregnant women and young children into the villages through Ambulatory Nurses.

Unexpectedly and unfortunately, the Government of Guatemala closed the PEC in late 2014 amidst a general breakdown of governmental services riven by mismanagement and corruption in high levels of government, including in the MSPAS [12]. While the termination of MSPAS’s PEC contracts for the Project Area negatively impacted final Project results (discussed further in Paper 3 [4], the initial success of the integration of the Project with the PEC/SIAS program provided an opportunity to investigate how CBIO+ and its implementation by Curamericas/Guatemala might feasibly and sustainably be adopted by the Government of Guatemala and supported by local communities in order to strengthen primary health care to rural areas. The evaluation focused on four primary areas: (1) advisability of integrating the CBIO+ Approach into the government’s rural health system, (2) staff knowledge of the CBIO+ Approach, (3) advantages, disadvantages and challenges of the CBIO+ Approach, and (4) proposed improvements to the CBIO+ Approach.

## Methods

The methods used for the research described here are described in detail in Paper 2 in this series [2]. To explore staff and key stakeholder perspectives on the strengths and weaknesses of the CBIO+ Approach, we followed a grounded-theory framework using self-administered open-ended questionnaires, key informant interviews, and focus group discussions (FGDs). In August 2013 (midway through the Project), the Project team conducted key informant interviews and two FGDs with both Project and MSPAS staff. In June 2015, to further understand key stakeholder perspectives and identify areas for improvement, an additional round of small group interviews were conducted with 2–4 Project staff and MSPAS staff as described below. Since the PEC/SIAS program had been terminated by the government in 2014, there were no PEC/SIAS staff to include in this round of interviews.

### Self-administered questionnaires

The Project utilized two different versions of a self-administered questionnaire to explore the staff’s perspectives on the strengths and weaknesses of the CBIO+ Approach. In August 2013, the questionnaire was completed by 25 Curamericas/Guatemala staff working in the three program municipalities, including two Institutional Facilitators, three Municipal Coordinators, an M&E Specialist, and 19 Level-2 Promoters. The questions focused on (1) staff knowledge of the CBIO+ Approach, (2) staff perceptions of the major advantages, disadvantages and challenges of the CBIO+ Approach, and (3) ways that the CBIO+ Approach could be improved. The results were then coded and tabulated on an Excel spreadsheet by a trained staff member using basic qualitative content analysis techniques. The respondents were stratified by job title and municipality in which the employee worked (San Sebastián Coatán, San Miguel Acatán and Santa Eulalia). Responses were sorted into thematic categories organized hierarchically. Each open-ended question had an average of 20 unique responses and these were sorted into 2–6 different thematic categories for each question.

### Focus group discussions and in-depth interviews with key informants

To augment the self-administered questionnaire results and expand on important themes that arose from them, we conducted five in-depth interviews and two FGDs with key personnel. The in-depth interviews, which also took place in August 2013, were designed to gather more information from the Municipal Coordinators, Care Group Supervisors (*Educadoras Supervisoras*), the Institutional Facilitators, the Level-1 Promoters, and the M&E specialist, most﻿ of whom had previously filled out the self-administered questionnaire. Further information about each role can be found in Paper 1 in this series [1]. All the individuals interviewed worked in the San Sebastián Coatán municipality. The interviews were designed and carried out by trained Curamericas/Guatemala staff.

Two FGDs were held in August 2013: (1) four MSPAS employees who were providing services to the San Sebastián Coatán municipality through PEC, and (2) one with six Curamericas/Guatemala Level-2 Promoters from San Sebastián Coatán. The FGD with PEC staff included two Level-2 Promoters, one nurse, and an information specialist. Because this group did not include any Curamericas/Guatemala Project staff and had not received any orientation on the CBIO+ Approach, these discussions focused on the relationship between PEC and Curamericas/Guatemala and opportunities for enhanced collaboration. The FGD with the Curamericas/Guatemala Level-2 Promoters included six from San Sebastián Coatán municipality, four of whom were very familiar with the CBIO+ Approach and two that had been recently hired. Each of these two FGDs were guided by the co-first author (JL) and led by two Curamericas/Guatemala staff members using a standard FGD approach [13].

An additional round of group interviews took place with key informants at the time of the Project’s final evaluation in June 2015 to meet the Project’s research goal of assessing and documenting the challenges and advantages of implementing the CBIO+ Approach and integrating it into the work of MSPAS. Interviewees were selected via convenience sampling from all three of the Project’s three﻿ municipalities: San Sebastián Coatán, San Miguel Acatán, and Santa Eulalia. There were 21 Level-2 Promoters employed by the Project included among the interviewees, with equal representation (*n* = 7) from each municipality﻿. Also, group interviews were conducted with 11 MSPAS staff (5 auxiliary nurses, 3 graduate nurses, 1 doctor, 1 secretary, and 1 counselor). There were 3–5 MSPAS staff from each of the three municipalities. Two to four interviewees participated in each of the interview sessions.

The co-first author (JL) worked with Curamericas/Guatemala staff to conduct the first round of interviews in 2013, the results of which were reported elsewhere [14]. The other co-first author (SM) worked with Curamericas/Guatemala staff to conduct the second round of interviews in 2015. JL and SM led the FGDs and the interviews. The results of the FGDs in 2013 informed the development of interview objectives and research questions in 2015. In 2013 there were two FGDs, one with four and the other with six participants, and in 2015 there were 8 separate group interview sessions, each with 3–5 key informants (with a total of 32 key informants participating).

Notes were taken in Spanish, translated and transcribed into English, and then analyzed. The sessions were recorded and the recordings were referred to when needed at the time of transcription. Categories of responses were identified and these were sorted into themes.

The updated Community Health Worker Assessment and Improvement Matrix (CHW AIM) toolkit [15] was applied in the analysis of the perspectives of the Project staff, staff of the PEC Program, and the local MSPAS staff with regard to: (1) advisability of integrating the CBIO+ Approach into the government’s rural health system, (2) staff knowledge of the CBIO+ Approach, (3) advantages, disadvantages and challenges of the CBIO+ Approach, and (4) proposed improvements to the CBIO+ Approach. Study participants spoke to seven of the 10 programmatic components of the toolkit: *role and recruitment, training, equipment and supplies, incentives, community involvement, data, and linkages to the national health system.* Below we﻿ cite these components in italics when the findings of our study correspond to one of these components.

## Results

### Advisability of integrating the CBIO+ Approach into the government’s rural health system

All groups interviewed provided positive feedback on the CBIO+ Approach and expressed a strong desire to continue using it. Community engagement (*community involvement*), the cascade model (*role and recruitment, training*), and community censuses (*data)* were cited  in support of integrating the CBIO+ Approach into the rural health system, while deficient health financing *(linkages to the national health system*) was an evident barrier.

Those interviewed – the Project staff, the staff of the PEC Program, and the local MSPAS staff – uniformly reported that the Project and its CBIO+ Approach provided important value to the health services through culturally relevant community participation that involved community leaders, community members, churches, *comadronas*, and local organizations to increase the coverage of basic and essential health services. Many of those interviewed stated that the Project’s community outreach components were more effective than the usual MSPAS clinic-based model for health care delivery because the former was grounded in generating communities’ trust and collaborating with them to improve their health. The Project’s recruitment and training of local women as Level-1 Promoters and Care Group Volunteers to go directly to the communities to meet and work with community members was cited in FGDs as a critical enabler of these foundational elements. Because the Project staff spoke the language of the communities and share the culture, there was an understanding and trust that would otherwise be impossible to create. One group interview respondent emphasized the value of reaching out into the community. She said:

It is important to visit the communities and to continue doing home visits. The MSPAS staff wait for community members to come to them for consultations, but this is not good because the people wait until they are in dire need to go to the MSPAS’s health posts.

– Level-2 Promoter, San Sebastián Coatán, 2015 small group interview

In recognition of this, all MSPAS staff explained that MSPAS did not currently have a sufficient supply of health workers to go to the communities and, even if it did, as outsiders the MSPAS staff would not be able to gain the support of community leaders and trust of the communities. As such, the MSPAS staff suggested increasing collaboration with the Project in order to gain the trust and support of the rural communities. For this reason, the Project staff stressed the importance of effective communication and coordination between the Project and the MSPAS. As one Project staff member said:

We need to all work together by integrating people from the MSPAS, the community members, and the Project so that it is a collaboration between all of us.

– Level-2 Promoter, Santa Eulalia, 2015 small group interview

Even in San Sebastián Coatán, where the MSPAS and Curamericas-Guatemala staff described a less collaborative relationship than in the other two municipalities, a MSPAS staff member acknowledged:

Someone must take on the work of the Health Educators [Level-2 Promoters] and Community Facilitators [Level-1 Promoters]. We must continue with where they left off. We have the responsibility to take care of these needs and to follow this model which has done great work in the communities.

- MSPAS Staff, San Sebastián Coatán, 2015 small group interview

While MSPAS staff in all the municipalities supported adopting the Project methodology, they cited a lack of support from higher-level MSPAS officials to employ community-level staff to do home visits—one of the most important aspects of the methodology identified by both the Level-2 Promoters and the MSPAS. At the end of the Project, the MSPAS in San Miguel Acatán and Santa Eulalia had begun to employ auxiliary nurses to replicate the work of the Project’s Level-2 Promoters, but budgetary constraints led to an insufficient number of nurses engaged in this activity. An MSPAS auxiliary nurse noted:

Integrating CBIO+ into their local health care system would be possible if the head coordinators thought more about the people of our communities….[This Project] is not an economic hardship. We can do very much with little money when there is drive and desire among the health workers to do humanitarian work.

- MSPAS Auxiliary nurse, Santa Eulalia, 2015 small group interview

Several MSPAS staff noted that the government should be investing more in health care. For example, one said:

We have very little money from the government…. The government spends only 2.5% [of its total budget] on health, so very little is invested in improving health. How are we going to improve the health with so few resources? It is a disaster. Here in the highlands is where there is very high mortality and we need more resources, but in fact fewer resources arrive here. It would be very helpful to be able to use these [Project] methodologies but it is very difficult for us to do so. Our [MSPAS] workers already have a lot to do and they are unable to do any more.

– MSPAS Staff, San Miguel Acatán, 2015 small group interview

Other respondents stated additional reasons for integrating the CBIO+ Approach into the MSPAS framework for primary health care delivery, including that it improves the accuracy of health data collection and it provides education on prevention and health promotion that can reduce the need for more costly health care services.

### Staff knowledge of the CBIO+ Approach

The detailed responses to the questionnaires, interviews, and FGDs show that the personnel working for Curamericas/Guatemala and MSPAS locally were very familiar with the CBIO+ Approach. Common responses demonstrated understanding of program components including roles of staff and community actors and the importance of recruitment of community members (*role and recruitment),* community participation throughout the approach (*community involvement*), training cascade (*training*), and community-level data collection to inform intervention priorities and monitor results (*data*). This is notable as the CBIO+ approach is a complicated conceptual framework and the local staff demonstrated an understanding of both its theory and operations.

Project and MSPAS personnel understood that the CBIO+ Approach is a primary health care framework that helps Project staff understand the most important community health needs and leads to the development of high-impact interventions that are contextually appropriate. In addition, project and MSPAS staff recognized that the CBIO+ Approach includes community participation at all levels, developing trust with communities, measuring the health situation, and monitoring programmatic progress.

**Fig. 1:**
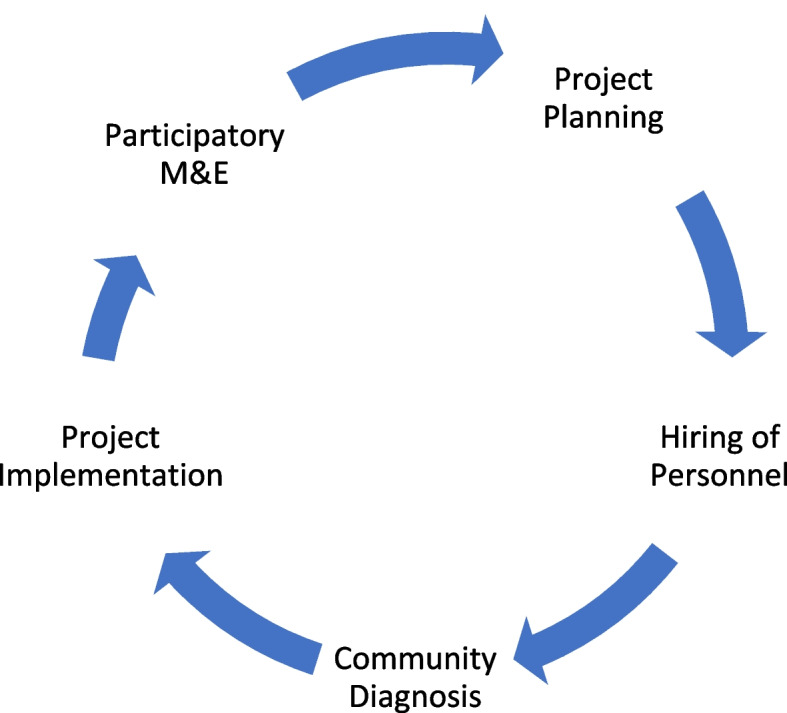
The cyclical progression of the CBIO+ Approach identified by Municipal Supervisors

Responses revealed familiarity with the individual components of the CBIO+ Approach, specifically CBIO and Care Groups. The three Municipal Coordinators correctly described the cyclical process involved in CBIO, from project planning to hiring of personnel to community diagnosis to project implementation, culminating in participatory M&E followed by repetition of the cycle (Fig. 1). They demonstrated a similar level of familiarity with the Care Group Approach. In addition to an understanding of the training cascade process, most of those interviewed recognized that the fundamental aim of Care Groups is to train beneficiaries on important health themes. The majority of staff also noted that vital events collection is an important component of the Care Groups, and that the health messages provided to the mothers had the potential to spread through social networks.

It can be difficult to describe the combination of the above methodologies into a single CBIO+ Approach, yet both the Curamericas/Guatemala staff and MSPAS staff demonstrated an understanding of the overall approach. They recognized that CBIO+ is founded upon building trust and involving all relevant parties, including beneficiaries and community leaders. Moreover, they identified that the essence of the approach is to identify the most important health needs in communities, develop an implementation plan with community participation, and use the Care Groups to educate mothers on these important health themes.

When asked about the principal tools used by the CBIO+ Approach, the Curamericas/Guatemala staff successfully listed the most important available tools. These included techniques for identifying the community and epidemiological priorities and for measuring and evaluating progress. To determine community priorities, the staff mentioned interviews and discussion groups with community leaders and beneficiaries. The tools mentioned by the respondents for determining the epidemiologic priorities included knowledge, practice and coverage (KPC) surveys, community censuses, vital events registration, verbal autopsies, and mini-KPC surveys (surveys with a small number of questions that were carried out at intervals between the baseline and endline KPC surveys). The tools mentioned for measuring Project results and health impacts included home visits, growth monitoring, and KPC surveys for measuring population coverage of key health interventions.

### Advantages, disadvantages and challenges of the CBIO+ Approach

There was consensus between the Project staff and MSPAS staff from each of the three municipalities on the advantages of the CBIO+ Approach. Project staff listed more disadvantages and challenges than MSPAS staff, but in general, MSPAS responses were similar to those of the Project staff in each municipality. Some of the greatest advantages were also described as challenges, including achieving community participation (*community involvement*) and conducting community censuses (*data*). As a disadvantage, insufficient remuneration (*incentives*) contributed to the challenges of high staff turnover. Advantages, disadvantages and challenges beyond the CHW AIM toolkit components were also described.

The most frequently mentioned advantages of the CBIO+ Approach mentioned by Project staff in the self-administered questionnaire were its highly participatory methods and its emphasis on community engagement to identify, design, and implement culturally appropriate effective and efficient health interventions. One respondent put it this way:

The methodology allows for the identification of community-level problems, the creation of a plan of action for each community, and provision of the opportunity for the population to participate at all steps.

- Municipal Supervisor, San Sebastián Coatán, 2013 self-administered questionnaire

Other advantages of the CBIO+ Approach mentioned by staff included its adaptability to different contexts, its impact-oriented nature, its empowering effect on participants to take actions to improve their own health, and the involvement of well-trained and well-supported Care Group Volunteers.

The most commonly mentioned challenge by many FGD participants in implementing the CBIO+ Approach was the high rate of migration of residents in and out of the communities. Project staff turnover was an additional challenge, requiring additional investments in training that were time and resource intensive. Participants also mentioned that the CBIO+ Approach can be difficult to implement, primarily due to the presence of severe poverty and economic instability as well as community discord, usually related to religious or social conflicts. Moreover, the respondents mentioned that the approach takes a great deal of time to carry out, it can be challenging to coordinate, and it does not create jobs in the community.

Another frequently reported challenge was the development and maintenance of the community censuses – especially the initial census, which takes place at the same time that the community mapping is underway. These are major tasks that require strong community collaboration. Another challenge noted was keeping the censuses updated and accurate.

Findings of both the self-administered questionnaires and the interviews included the challenge of achieving high levels of participation of mothers in meetings with the Care Group Volunteers. After each meeting of Care Group Volunteers with a Level-1 Promoter, each Care Group Volunteer would be responsible for conveying the messages learned to the mothers in her catchment area. This could be achieved by meeting with each mother individually in her home or by calling together these women for a meeting. Given that in many communities homes were quite dispersed, it was convenient for the Care Group Volunteers to meet with her mothers in a group, which was referred to as a *Grupo de Autocuidado* (Self-help Group). Unfortunately, most Care Group Volunteers reported rates of 67–85%, rarely reaching 100% of the beneficiaries. There were a few communities that had poor overall participation. The respondents reported that the most common reason for non-participation was the *machismo* culture in the communities, with husbands/partners often preventing women’s attendance at the Self-Help Group meetings. Other barriers to participation in the Self-Help Group meetings included the time of day of the meetings (afternoons were better than mornings), the presence of housework that could not be neglected, apathy among the mothers, and a lack of familiarity with the Project.

Other challenges to implementing the CBIO+ Approach mentioned by the respondents included attaining community trust, communicating with the community, dealing with social upheaval, and adjusting to community in- and out-migration. The final implementation challenge mentioned was Project staff turnover. Because of the useful skills attained by Project staff and their low salaries, a number of them were recruited by other organizations at mid-stream during the implementation of the Project.

### Proposed improvements to the implementation of the CBIO+ Approach

Study participants proposed several improvements to the implementation of the CBIO+ Approach. These suggestions address many of the challenges identified above. They also link to the functionality of programmatic components included in the CHW AIM toolkit including increasing community participation (*community involvement*)*,* ensuring an adequate supply of commodities directly or through referrals (*equipment and supplies, linkages to national health system*), reducing the burden of travel and improving service delivery coordination *(linkages to the national health system)*, making data collection more efficient (*data*), and adequately remunerating all volunteers and staff according to their responsibilities and skill level (*incentives*).

#### Improving the participation of mothers in the Self-Help Groups

The staff suggested involving men more in the Project, as this would both increase participation and facilitate familial behavior change. This could be in the form of formal heath education for the males, or simply making them aware of the current health situation in their community and in their family so that they understand the importance of allowing their wives to participate. Another suggestion was to have more meetings in the afternoon when the women have less housework and their husbands are more likely to allow them to leave. This is consistent with findings from the Project’s Care Group attendance records which showed that afternoon meetings were better attended than the morning meetings. In an FGD, the Level-2 Promoters emphasized the importance of improving women’s self-esteem as a way to improve participation. An additional explanation for low participation of mothers in the Care Group meetings relates to the provision of medication. One of the Level-2 Promoters succinctly described the situation when she said,

They [the mothers] ask ‘did you bring medicine?’ and we have to say no. We are in a very, very poor area and the mothers tell us ‘my child is sick…and you cannot cure him.’

Level-2 Promoter, San Sebastián Coatán, 2013 Focus Group Discussion

While the Project made referrals to PEC staff at government health posts, which can be successful, many of the women found this inadequate as they did not have the time or access to a vehicle that would be needed to travel an hour or more to the nearest health post. As a result, some became frustrated with the Project, and this may have affected their participation. This sentiment was affirmed by the Level-2 Promoters, who also pointed out that participation is high when the Project provides commodities. One said,

Right now, we are giving a nutritional supplement ﻿[for children], and more of the women are participating in vaccinations and other health activities. Level-2 Promoter, San Sebastián Coatán, 2013 Focus Group Discussion

Some of the interviewed staff argued that if they were able to devote more time to the communities, they would be able to develop stronger relationships with mothers and provide better education to them, and this, in turn, would result in greater participation. From this perspective, there were two potential solutions that were frequently mentioned: (1) decreasing the number of communities assigned to each Level-2 Promoter, and (2) providing transportation support to the Level-2 Promoters. (Each Level-2 Promoter was responsible for Care Groups in approximately seven communities). Hiring additional Level-2 Promoters would have allowed each one to spend more time in each community. The second proposed solution was related to the time it takes to travel to the communities. It can take a Level-2 Promoter, who was almost always a woman, more than 2–3 h to arrive at a community by foot, and this is often the only option. There was an﻿ overall shortage of vehicles available to the program (the Project had only one vehicle), and the Level-2 Promoters did not know how to ride a motorcycle. The Level-2 Promoters often spent more time walking than they did working in the communities. Potential solutions to this problem offered by the respondents included purchasing more vehicles and motorbikes, training the Level-2 Promoters to ride motorbikes, and coordinating better the use of available transport with the MSPAS and ADIVES staffs.

The final frequently mentioned reason for women not participating was a lack of trust in the Project among community members. Specifically, participants mentioned that in some communities, people doubted the intentions of the Level-2 Promoters and believed that they were collecting data for reasons other than improving health. This affected their willingness to participate. Moreover, it was mentioned that the Project staff were still not permitted in some communities, and there had been threats of physical violence should they come. In response to this problem, the staff mentioned that the Project should focus on building greater trust with community leaders. The final suggestion was to introduce the Project staff at community meetings to familiarize the community with the Project.

#### Strengthening coordination and communication

Improving coordination with other groups was mentioned by interviewees. In interviewing the PEC staff it was clear that the relationship with Curamericas/Guatemala was very good except for coordination of transport. According to the PEC staff, the two organizations did plan together *which* communities will be visited, but they did not plan together *when* this would happen. The result was that they might visit the same community a day apart, which turned out to be both a missed opportunity for decreasing travel time of Project staff (since the PEC staff and even some MSPAS staff had a vehicle) as well as a frustration for the community members who had to change their daily routines and responsibilities to accommodate the visits.

Communication was a further point of concern for the staff who were interviewed. One of the questions on the self-administered questionnaire asked how well the Project’s communication chain functioned and what its weakest links were. Overall, respondents thought it worked well, but there were a few challenges noted. The two most frequently cited weak links were between the Care Group Volunteers (called *Comunicadoras en Salud*, or Health Communicators) and the paid lower-level Project staff (Level-1 and Level-2 Promoters) as a result of some of the paid staff not being able to speak the local dialect. This led to difficulties in establishing trust with mothers, affecting their participation in Care Group activities.

#### Strengthening project activities

Another area in which the staff suggested improvements concerned the community censuses, specifically updating them on a regular basis. One suggestion for improving the census process was to hire a temporary employee to update the census regularly. This would have freed up time for the Level-2 Promoters to give more attention to education and to addressing undernutrition. A Care Group Supervisor mentioned that if she could change one thing moving forward, she would deemphasize all activities not directly related to education and undernutrition, which she believed to be the two most critical needs in the community.

Another area that the Curamericas/Guatemala staff described as challenging was changing the behavior of mothers in the face of apathy and a lack of interest in changing. One participant mentioned that the Positive Deviance exercises that the Project had carried out with the communities had been helpful in changing behaviors:

For example, they [the communities] say that colostrum is bad. They say it is dirty, that it should not be used, so they throw it out [and give the newborns other liquids]. And we, the team, arrive and tell them that it is good…it is a contradiction. If you find a person or a mother who exclusively breastfeeds, you can use her as an example. You can say, ‘here is a mother who exclusively breastfeeds, and you can see that her child is large and healthy.’ This is an example I have used. To achieve behavior change, you have to use solutions from the communities themselves.

Level-2 Promoter, San Sebastián Coatán, 2013 Focus Group Discussion

The Level-2 Promoters also mentioned that one must have a great deal of patience since behavior change takes time. It is also important, they said, to improve the self-worth of the women; only when they value their own lives will they be interested in changing their behaviors.

The Level-2 Promoters suggested strengthening linkages between CBIO+ and the MSPAS health services to create referral pathways for community members needing medicine or treatment. This stemmed from the communities’ frustration that the Project did not provide medicines or medical treatment. The Level-2 Promoters reported educating families on emergency medical transportation and seeking secondary and tertiary care at formal health centers, but these health centers were not always able to meet patients’ needs because of limited resources. This caused them to lose faith in both the Project and MSPAS.

#### Staff strengthening

Finally, the Project staff mentioned a need to improve staff motivation and efficiency. The most common suggestion was to improve the incentive structure for both the Level-1 Promoters and the Care Group Volunteers. Almost all Project staff informants mentioned that it would have been prudent to provide an incentive for the Care Group Volunteers. The Care Group Volunteers were not paid, and this most certainly affected the quality of their work. The respondents also noted that the Care Group Volunteers performed much of the same work as Level-1 Promoters, yet the Level-1 Promoters were paid a small stipend while the Care Group Volunteers were not, leading to a perception of unfairness among the Care Group Volunteers.

Nearly all Project staff recognized that the work of the Care Group Volunteers was the foundation of the Project, and the staff mentioned several ways in which they could have been incentivized. One way could have been paying them with a "basic basket" that included healthy foods. Even providing snacks for the Care Groups when they met could have been a small encouragement. It was also mentioned that it would have been important to remind the Care Group Volunteers constantly that the work they did was extremely important because of its benefits for the community and that it was greatly appreciated.

Regarding the incentive structure for the Level-1 Promoters, participants suggested that they should have received more pay or other additional incentives. Nearly every staff member expressed the extreme indispensability of the Level-1 Promoters to the work of the Project. It was suggested that an increase in pay would have rewarded them for their high quality of work and decreased turnover. A number of Level-1 Promoters were able to obtain better employment elsewhere. Because Level-1 Promoters required several months of on-the-job training, several respondents reported that the resignation of Level-1 Promoters to take other jobs was a major setback for the Project. Potential solutions that were mentioned by those interviewed included compensating Level-1 Promoters more or paying them by the hour (including overtime), or providing them with non-financial incentives such as additional training.

### Implementation of Project improvements

Many of the challenges identified above are not intrinsic to the CBIO+ Approach and are specific to the rural Guatemalan context. As such the Project team was able to adapt and incorporate improvements into Project implementation address some of these issues, many of which became less acute at the time of subsequent interviews in 2015. For example, it became apparent that community participation had increased since the FGDs in 2013. Significant efforts had been made to improve community trust and participation, including holding community assemblies that were open to all community members; these were felt to be the primary reason for the increase in participation. As one staff member noted:

There were no assemblies at the beginning. We only talked with community leaders but we now have meetings with the people so that everyone can come…. In this way…all [community members] are able to understand.

– Level-2 Promoter, San Sebastián Coatán, 2015 small group interview

The increase in community awareness of the interventions provided by Curamericas/Guatemala and the intended causal pathway to improved health, coupled with increased acceptance by community leaders, led to an improvement in communication with local communities. Similarly, the recruitment of local women as Care Group Volunteers contributed to increased community participation and trust between the community and Project.

## Discussion

The information arising from numerous interviews and FGDs with Project staff as well as with local MSPAS staff indicates that there is broad support for the incorporation of the CBIO+ Approach into the local primary health care system of the Project Area and beyond. The benefits of broader community participation and community outreach were widely appreciated by those interviewed. Notable challenges mentioned by the respondents included the time-consuming nature of the approach as well as the difficulty of implementation when there are high rates of out- and in-migration as well as when there is conflict in the communities. Therefore, keeping the census accurate can be a challenge. Although participation in Care Groups reached 67–85% of mothers with young children, there were challenges including inconvenient timing of the meetings of Self-Help Groups (*Grupos de Autocuidado*) and, most importantly, resistance from husbands/partners to their participation. *Machismo* reinforces male dominance at the expense of empowerment of women and their self-esteem, as discussed in Papers 7 [7] and 8 [8].

Overall, the challenges and limitations identified were more focused on contextual issues rather than on the CBIO+ Approach itself. However, one criticism of the Care Group model made by the respondents is worth emphasizing – namely the inability of the Care Group Volunteers and Level-1 Care Group Promoters to treat childhood illness. The home visitation done by Care Group Volunteers focused on health monitoring and health education, including recognition of danger signs for which referral is needed. However, in such an isolated locale as the Project Area, accessing medical care is challenging. Providing the Level-1 and Level-2 Promoters with training in Integrated Community Case Management of Childhood Illness (which community health workers throughout the world now receive [16]), would have been an important addition to the CBIO+ Approach. Unfortunately, the government’s policy did not permit this at the time of Project implementation (and it still does not). As those interviewed mentioned, providing commodities (such as supplements of food or vitamins) to mothers might have improved Care Group participation.

Building stronger referral systems was a widespread need mentioned by the respondents, especially since the capacity to provide acute curative care at the community level is so limited. Community-level staff need to know where to refer patients with acute illness so that they are not sent to a facility that cannot provide appropriate care, leading to loss of confidence in the health care system.

Increasing the numbers of paid staff and providing them with better transportation support could have also improved the implementation of Project activities. Mothers’ lack of trust in the Project was an understandable challenge given the long history of oppression and conflict in the Project Area (described in Paper 1 [1]).

The insights provided by the respondents suggest that the Project could have benefitted by relying more on home visits from the Care Group Volunteers than on expecting mothers to meet in Self-Help Groups. Care Group Volunteers were expected to contact the 10–15 mothers they were responsible for between meetings of the Care Groups. Self-help Groups were envisioned as a way for Care Group Volunteers to contact their mothers in a more efficient manner: instead of visiting every home, the Care Group Volunteer could meet with their mothers as a group and (presumably) go to the homes of the mothers who did not come to the meeting. However, because, according to Project staff, as many as one-third of mothers did not participate in these Self-Help Groups, participation of all mothers could have increased if the Care Group Volunteers regularly visited the homes of all the mothers for whom they were responsible. This could also have helped to overcome distrust of the Project that mothers (and their husbands/partners) may have had.

Among its many benefits, CBIO+ delivers health education and connects beneficiaries to health care services that are specific to the most prominent health needs of the community while at the same time fostering local ownership and empowerment [17–19]. Of course, if the Project had had more money it could have had more vehicles and motorcycles, more and better qualified staff with higher pay, and less staff turnover. Nonetheless, given the tight budget that the Project had to work with (described further in the following, final paper in this series [20]), the short period of implementation (44 months in Area A and 20 months in Area B), and the Project’s achievements documented in this series of papers, a highly effective compromise was achieved.

The complementarity of the CBIO+ Approach with the government’s PEC Program was readily apparent during the interviews that took place mid-point in the Project’s implementation (when the PEC Program was still in operation). At the time of the interviews carried out during the final evaluation, the PEC Program had been cancelled by the government, and its value at that time was even more widely appreciated since the PEC Program had been sending nurses to visit each community at least once a month to provide health services. The Community Birthing Centers were beginning to provide some of the services previously provided by PEC nurses since the Birthing Centers had become equipped with small pharmacies and had become places for provision of antenatal care, delivery and post-partum care, treatment of sick children, and contraceptive distribution.

The need for long-term financing was widely recognized if the CBIO+ Approach were to become a sustainable addition to the local health system. In discussions with staff and others working in health care there, many considered that the municipalities themselves could help to bridge the gap in needed finances. Building the political will to make this happen remained a challenge at the conclusion of the Project.

### Limitations of this study

There are several limitations to this study. First, the transcripts of the interviews were coded and analyzed by a single person. Having a second person involved in these activities would have added additional robustness to the findings. Even so, similarities in findings from the data collected in 2013 and in 2015 offer additional validation to the findings. Second, the convenience sampling of willing and available Project staff and MSPAS staff might have introduced a self-selection bias. Of note, the 2013 FGDs involved respondents from one Project municipality, whereas the respondents from the 2015 interviews involved respondents from all three Project municipalities. Another limitation is that this study was designed before the Project had decided to include the Birthing Centers as part of the conceptual CBIO+ Approach, so there were no questions included that pertained to the Birthing Centers. Also, due to language, travel, staffing and time constraints, beneficiaries of the Project (e.g., mothers, families) were not included as informants for the study; their perspectives undoubtedly would have strengthened the results. Finally, there was the risk of social desirability bias: those who worked as employees and volunteers with the Project may have avoided mentioning some of their criticisms of the Project and its CBIO+ Approach.

## Conclusion

The many staff members of the Project and of the MSPAS who participated in this study considered the integration of the CBIO+ Approach into the existing MSPAS local health care delivery system as highly desirable. Many of the challenges and recommendations mentioned by those interviewed were specific to the Guatemalan context and were not intrinsically related to the CBIO+ Approach. For example, challenges with the *machismo* culture are relevant to any project working in the area. There are, however, several lessons from this assessment that may help improve the CBIO+ Approach regard of the local context.

As mentioned in other papers in this series, the CBIO+ Approach calls for program evaluations approximately every 4–5 years, but it may also be helpful to include ongoing implementation research similar to this study as an integral part of the approach itself. This would allow for a response to contextually specific concerns early on, thereby increasing the flexibility and therefore the effectiveness of projects utilizing CBIO+ . Second, it may have been advisable to include some activities specifically for men. This could have had many benefits, including building greater trust in the Project, increasing participation in Care Groups, and obtaining greater acceptance of proposed behavior changes.

The findings of this assessment, particularly the quoted comments of the MSPAS staff, are even more relevant today than when the Project ended in 2015. At present, the Guatemalan health system is primarily focused on facility-based, higher-level care. The kinds of activities that the PEC Program and Curamericas/Guatemala provide are not valued to the degree that they should be. Consequently, there is persistent marginalization of rural, indigenous people, resulting in the glaring health disparities they suffer. Additionally, there is a continued trend to privatization, with out-of-pocket expenses an increasingly greater share of per capita health expenditures, to the detriment of poor and rural populations. Greater commitment to community health at the national level is needed to realize the advantages of and the recommended changes to the CBIO+ Approach described by the Project staff and local MSPAS staff.

## Data Availability

All of the Project reports, de-identified data, as well as publications about the Expanded CBIO+ Approach cited in this article are available from the corresponding author on request.

## Abbreviations

CBIO: Census-Based, Impact-Oriented Approach
CBIO+: Combination of the CBIO Approach with Care Groups and Community Birthing Centers (*Casas Maternas Rurales*)
FGD: Focus group discussion
KPC: Knowledge, practice, and coverage (household survey)
M&E: Monitoring and evaluation
MSPAS: *Ministerio de Salud Pública y Asistencia Social* (Ministry of Public Health and Social Welfare)
PEC: *Programa de Extensión do Cobertura* (Extension of Coverage Program)
SIAS: *Sistema Integral de Atención en Salud* (Integrated System of Health ﻿Services of the Ministry of Public Health and Social Welfare)

## References

[1] Valdez M, Stollak I, Pfeiffer E, Lesnar B, Leach K, Modanlo N, Westgate C, Perry H. Reducing inequities in maternal and child health in rural Guatemala through the CBIO+ approach of Curamericas: 1. Introduction and project description. Int J Equity Health. 2023;21:Suppl 2. 10.1186/s12939-022-01752-y 10.1186/s12939-022-01752-y PMC9976357 36855139

[2] Perry H, Valdez M, Blanco S, Llanque R, Martin S, Lambden J, Gregg C, Leach K, Olivas E, Mufoletto B, et al. Reducing inequities in maternal and child health in rural Guatemala through the CBIO+ approach of Curamericas: 2. Study site, design and methods. Int J Equity Health. 2023;21:Suppl 2. 10.1186/s12939-022-01754-w. 10.1186/s12939-022-01754-w PMC9976360 36855098

[3] Blanco S, Valdez M, Stollak I, Westgate C, Herrera A, Perry H. Reducing inequities in maternal and child health in rural Guatemala through the CBIO+ approach of Curamericas: 3. Expansion of population coverage of key interventions. Int J Equity Health. 2023;21:Suppl 2. 10.1186/s12939-022-01755-9. 10.1186/s12939-022-01755-9 PMC9976355 36855129

[4] Perry H, Stollak I, Llanque R, Blanco S, Jordan-Bell E, Shinhelm A, Westgate C, Herrera A, Valdez M. Reducing inequities in maternal and child health in rural Guatemala through the CBIO+ approach of Curamericas: 4. Nutrition-related activities and improvements in childhood nutritional status. Int J Equity Health. 2023;21(Suppl 2). 10.1186/s12939-022-01756-8. 10.1186/s12939-022-01756-8 PMC9973244 36855101

[5] Perry H, Stollak I, Llanque R, Okari A, Westgate C, Shindhelm A, Chou V, Valdez M. Reducing inequities in maternal and child health in rural Guatemala through the CBIO+ approach of Curamericas: 5. Mortality assessment. Int J Equity Health. 2023;21(Suppl 2). 10.1186/s12939-022-01757-7. 10.1186/s12939-022-01757-7 PMC9976377 36855128

[6] Olivas E, Valdez M, Muffoletto B, Wallace J, Stollak I, Perry H. Reducing inequities in maternal and child health in rural Guatemala through the CBIO+ approach of Curamericas: 6. Management of pregnancy complications at Community Birthing Centers (Casas Maternas Rurales). Int J Equity Health. 2023;21(Suppl 2). 10.1186/s12939-022-01758-6. 10.1186/s12939-022-01758-6 PMC9976365 36855147

[7] Gregg C, Valdez M, Stollak I, Martin S, Story W, Perry H. Reducing inequities in maternal and child health in rural Guatemala through the CBIO+ approach of Curamericas: 7. The empowering effect of Care Groups. Int J Equity Health. 2023;21:Suppl 2. 10.1186/s12939-022-01759-5. 10.1186/s12939-022-01759-5 PMC9976358 36855142

[8] Stollak I, Valdez M, Storey W, Perry H. Reducing inequities in maternal and child health in rural Guatemala through the CBIO+ approach of Curamericas: 8. Impact on women’s empowerment. Int J Equity Health. 2023;21:Suppl 2. 10.1186/s12939-022-01760-y. 10.1186/s12939-022-01760-y PMC9976544 36855052

[9] Lao Pena C. Improving Access to Health Care Services through the Expansion of Coverage Program: The Case of Guatemala. UNICO Studies Series; No. 19. Washington DC: World Bank; 2013. https://openknowledge.worldbank.org/handle/10986/13283. Accessed 4 June 2022.

[10] MCSP. Guatemala Country Program: Summary. Maternal and Child Survival Program. Washington, DC: United States Agency for International Development; 2017. https://www.mcsprogram.org/wp-content/uploads/2017/04/Guatemala-Country-Summary-March-2017.pdf. Accessed 4 June 2022.

[11] Perry H, Morrow M, Borger S, Weiss J, DeCoster M, Davis T, Ernst P (2015). Care Groups I: An Innovative Community-Based Strategy for Improving Maternal, Neonatal, and Child Health in Resource-Constrained Settings. Glob Health Sci Pract.

[12] Call C, Hallock J (2020). Too Much Success? The Legacy and Lessons of the International Commission Against Impunity in Guatemala.

[13] Kitzinger J (1995). Qualitative research. Introducing focus groups. BMJ.

[14] Lambden J (2014). Linking of the Census-based, Impact-Oriented Methodology with Care Groups: An Approach to Effective Primary Health Care Programming (Master's Degree Capstone Essay).

[15] Ballard M, Bonds M, Burey J, et al. Updated Program Functionality Matrix for Optimizing Community Health Programs: Community Health Worker Assessment and Improvement Matix (CHW AIM). New York: UNICEF; 2018. https://www.unicef.org/media/58176/file. Accessed 4 June 2022.

[16] Boschi-Pinto C, Labadie G, Dilip TR, Oliphant N, Dalglish SL, Aboubaker S, Agbodjan-Prince OA, Desta T, Habimana P, Butron-Riveros B (2018). Global implementation survey of Integrated Management of Childhood Illness (IMCI): 20 years on. BMJ Open.

[17] Beracochea E, Valdez M, Perry H, Nancy N, Stracuzzi G. Curamericas Guatemala: Census-Based, Impact-Oriented Child Survival Project 2002-2007: Final Evaluation Report. Raleigh: Curamericas Global; 2007.

[18] Shanklin DS, Sillan D. The Census-Based, Impact-Oriented Methodology: Resource Guide for Equitable and Effective Primary Health Care. Raleigh: Curamericas Global; 2005. https://pdf.usaid.gov/pdf_docs/PBAAC582.pdf. Accessed 4 June 2022.

[19] Laughlin M. The Care Group difference: a guide to mobilizing community-based volunteer health educators. 2004. http://www.coregroup.org/storage/documents/Resources/Tools/Care_Group_Manual_Final__Oct_2010.pdf.

[20] Perry H, Stollak I, Valdez M. Reducing inequities in maternal and child health in rural Guatemala through the CBIO+ approach of Curamericas: 10. Summary, cost-effectiveness, and broader policy implications. Int J Equity Health. 2023;21:Suppl 2. 10.1186/s12939-022-01762-w. 10.1186/s12939-022-01762-w PMC9976361 36855130

